# Nitrogen Supply and Leaf Age Affect the Expression of TaGS1 or TaGS2 Driven by a Constitutive Promoter in Transgenic Tobacco

**DOI:** 10.3390/genes9080406

**Published:** 2018-08-10

**Authors:** Yihao Wei, Aibo Shi, Xiting Jia, Zhiyong Zhang, Xinming Ma, Mingxin Gu, Xiaodan Meng, Xiaochun Wang

**Affiliations:** 1 Collaborative Innovation Center of Henan Grain Crops, Henan Agriculture University, Zhengzhou 450000, China; yc_yihao@163.com (Y.W.); zyong1988@126.com (Z.Z.); xinmingma@126.com (X.M.); mengxd13@163.com (X.M.); 2 Department of Biochemistry and Molecular Biology, College of Life Science, Henan Agriculture University, Zhengzhou 450000, China; jinbangxiangge@163.com (A.S.); xitingjia_005@163.com (X.J.); gumingxin3@163.com (M.G.); 3 State Key Laboratory of Wheat and Maize Crop Science in China, Henan Agriculture University, Zhengzhou 450000, China

**Keywords:** tobacco, overexpression, glutamine synthetase 1, glutamine synthetase 2, expression regulation, nitrogen metabolism

## Abstract

Glutamine synthetase (GS) plays a key role in nitrogen metabolism. Here, two types of tobacco transformants, overexpressing *Triticum aestivum* GS1 (TaGS1) or GS2 (TaGS2), were analysed. Four independent transformed lines, GS1-TR1, GS1-TR2, GS2-TR1 and GS2-TR2, were used for the nitrogen treatment. Under nitrogen-sufficient conditions, the leaves of GS2-TR showed high accumulation of the *TaGS2* transcript, while those of GS1-TR showed a low *TaGS1* transcript levels. However, compared with nitrogen-sufficient conditions, the *TaGS1* transcript level increased in the leaves under nitrogen starvation, but the *TaGS2* transcript level decreased. In addition, the *TaGS1* and *TaGS2* transcript levels were highest in the middle leaves under nitrogen-sufficient and starvation conditions. These results show that nitrogen supply and leaf age regulate *TaGS* expression, even when they are driven by a super-promoter. Additionally, in regard to nitrogen metabolism level, the lower leaves of the GS1-TR exhibited lower NH_4_^+^ and higher amino acid contents, while the upper leaves exhibited higher amino acid, soluble protein and chlorophyll contents. The leaves of the GS2-TR exhibited lower NH_4_^+^ but higher amino acid, soluble protein and chlorophyll contents. Given the role that GS isoforms play in nitrogen metabolism, these data suggest that *TaGS1* overexpression may improve nitrogen transport, and that *TaGS2* overexpression may improve nitrogen assimilation under nitrogen stress.

## 1. Introduction

Nitrogen is an important macronutrient that is a limiting factor in the production and yield of crops [1]. Nitrogen is an important limiting factor for the yield and quality of wheat and requires large quantities of nitrogenous fertilisers to attain maximal growth and productivity [2]. To increase crop production in line with human population growth, nitrogen fertilisers are being applied excessively, leading to severe nitrogen pollution globally [3,4]. Therefore, there is a need to improve nitrogen use efficiency (NUE) to make agriculture more sustainable [5].

Glutamine synthetase (GS; EC 6.3.1.2) catalyses the conversion of NH_4_^+^ into glutamine, which serves, together with glutamate, as a nitrogen donor for the biosynthesis of all other amino acids and then other nitrogenous compounds, such as protein, chlorophyll, and nucleotides [6]. For this reason, GS plays a key role in nitrogen assimilation in higher plants [2,7,8]. 

GS is expressed in the form of two isoforms with different subcellular localisation: the cytosolic isoform (GS1) and the chloroplastic isoform (GS2) [9]. GS1 (38–40 kDa) is encoded by 3–5 nuclear genes, whereas GS2 (42–45 kDa) is encoded by one nuclear gene (Bernard et al., 2008) [10]. GS2 is the predominant isozyme in leaf mesophyll cells, it plays a role in assimilating NH_4_^+^ derived from photorespiration and nitrate reduction [11]. GS1 has multiple metabolic functions, such as assimilating ammonia into glutamine for transport and distribution throughout the plant, reassimilation of the NH_4_^+^ generated from various catabolic processes during leaf senescence, and assimilation of ammonium derived from uptake from soil and nitrate reduction in roots [12,13,14,15,16,17]. Roots are central to the acquisition of water and mineral nutrients, including nitrogen, and the GS1 in roots plays an important role in nitrogen absorption and assimilation, which affects the NUE of plants [18,19]. 

Since GS have important functions in nitrogen assimilation, the GS has been investigated in many cases with the goal of improving crop NUE [2,17,20,21]. Quantitative trait locus analyses performed in several cereal species have suggested that genotypic differences in NUE can partly be explained by GS loci [5]. Kichey, Heumez, Pocholle, Pageau, Vanacker, Dubois, Le Gouis and Hirel [2] found that there was a strong relationship among total N, chlorophyll, soluble protein, ammonium, amino acids and GS activity in wheat. In previous study, we found that the regulation of GS isozymes may promote flow strength and enhance NUE by a complex carbon-nitrogen metabolic mechanism [22]. Therefore, one potential way to improve NUE is to enhance GS enzyme activity in plants. However, previous research showed that the overexpression of GS may have different effects on the observable phenotype and nitrogen metabolism [5,23].

Many studies have reported increased GS activity following overexpression of *GS1* genes [24,25,26,27]. However, the phenotypes of the transgenic plants were not completely consistent, and negative effects on plant productivity have even been recorded. For example, tobacco overexpressing the *GS1* gene from pea exhibited higher vegetative biomass under conditions of nitrogen starvation and high nitrogen [27], but tobacco expressing the *GS1* gene from alfalfa showed better growth only under nitrogen starvation [25]. Transgenic wheat lines overexpressing the *GS1* showed significantly higher grain yield and grain nitrogen content [24]. However, Thomsen, Eriksson, Møller and Schjoerring [5] have not been able to reproduce these results in their laboratory using the same wheat lines. In rice, overexpression of the *Oryza sativa GS1* (*OsGS1*) gene led to improvement of spikelet yield in plants grown in the growth chamber under non-limiting conditions [28] and resulted in increases in soluble protein and nitrogen content when plants were grown in a controlled environment [29]; however, the grain yield significantly decreased when the transgenic lines were grown in greenhouse or under field conditions [28,29]. In *GS2*-overexpressing plants, the total amino acid content was improved in transgenic non-heading Chinese cabbage overexpressing the *Brassica campestris GS2* (*BcGS2*) [30]. In addition, overexpression of the *Nicotiana tabacum GS2* (*NtGS2*) driven by the rbcS promoter enhanced the growth of tobacco seedlings [31]. However, growth and vegetative biomass were all significantly reduced in transgenic tobacco overexpressing the *GS2* gene from pea [27]. Among the factors causing differential responses were the transformation plant, the specific donor gene, nitrogen supply and growth conditions.

In cells, the inorganic nitrogen assimilation process that GS participates in consumes a substantial amount of energy [5,32]; therefore, GS must be tightly regulated at the gene, transcript and protein levels. In wheat, nitrogen starvation improved *GS1* expression but led to a reduction in *GS2* expression in leaf of 15-day-old plants [33]. After heading stage, the expression level of GS1 in leaf was great higher in low nitrogen treatment than that in high nitrogen treatment [34]. During leaf senescence, nitrogenous compounds are used as nutrient sources for the building of new organs and for grain-filling in cereals [20]. In leaf, the main source of nitrogen for remobilisation is chloroplasts, so the expression of chloroplastic isoform, GS2, decreases with leaf ageing, while the cytosolic isoform, GS1, is responsible for the re-assimilation of ammonium in old leaves, the expression of GS1 increased in the mesophyll of senescing leaves [10,34,35,36]. Based on the above reports, we hypothesised that *Triticum aestivum* Glutamine synthetase (*TaGS*) expression may follow the same rule in *GS* transgenic plants as it does in wild type (WT) plants, depending on the specific *GS* gene, plant developmental stage and environmental conditions, which can affect nitrogen metabolism and result in different phenotypes. A better insight into these mechanisms is necessary if attempts to use overexpressing *TaGS* to improve wheat NUE are to be successful.

Highly active constitutive promoters have been widely used in many studies; in most cases, the gene, driven by a constitutive promoter, is expressed constitutively. However, the *GS1* transcript, driven by the cauliflower mosaic virus (CaMV) 35S promoter, decreased significantly when transgenic alfalfa was grown under high-nitrogen conditions [37]. Maybe the driven efficiency of CaMV 35S promoter is not sufficient under high-nitrogen conditions. The super-promoter is a synthetic promoter consisting of a trimer of the octopine synthase transcriptional activating element linked to the mannopine synthase 2’ (*mas2’*) activator-promoter region [38]. In tobacco leaves, the super-promoter is approximately 156-fold stronger than the CaMV 35S promoter [39]. The super-promoter has routinely and effectively been used to drive both transient and stable transgene expression in tobacco and other plants [39,40,41,42]. Therefore, we chose the super-promoter to drive the *TaGS1* and *TaGS2*, ensuring that the efficiency is sufficient. 

In this study, we initially investigated the expression patterns of *TaGS1* and *TaGS2* driven by the super-promoter in the leaf of different rank and roots of tobacco growing under nitrogen sufficiency and nitrogen-starvation conditions. Based on the transcript levels, polypeptide abundances and activities of *TaGS1* and *TaGS2* transformants, we examined the effect of *TaGS1* or *TaGS2* overexpression on nitrogen metabolism and plant growth to determine whether GS overexpression can improve nitrogen use efficiency. 

## 2. Materials and Methods 

### 2.1. Plasmid Construction

The cDNA of *TaGS1* and *TaGS2* were obtained from wheat (*T. aestivum* L.) cultivar Yumai 49 in our laboratory previously [43]. Modified pCAMBIA 1300 [40] was kindly provided by Dr. Hairong Zhang (China Agriculture University, Beijing, China). The CDS (Coding Sequence) of *TaGS1* and *TaGS2* were amplified using specific primers (Sangon Biotech Co., Ltd., Shanghai, China) (Table S1). The PCR was performed on a T100 Thermal cycler (Bio-Rad, Hercules, CA, USA), and the conditions were as follows: initial denaturation at 94 °C for 2 min, 30 cycles at 94 °C for 45 s, 70 °C for 1 min and 72 °C for 70 s, with a final elongation at 72 °C for 10 min. The PCR products were respectively cloned into the modified pCAMBIA 1300 plasmid. The recombinant vectors were constructed using ClonExpress One Step Cloning Kit (Vazyme Biotech Co., Ltd., Nanjing, China). The *TaGS1* or *TaGS2* CDS was cloned as a *Spe*I-*Xba*I fragment downstream of the super-promoter [38] in the modified pCAMBIA 1300 binary vector (Figure S1). The recombinant plasmid was introduced into *Agrobacterium tumefaciens* strain GV3101 (provided by Dr. Hairong Zhang), which was used to transform tobacco. 

### 2.2. Plant Transformation and Growth Conditions

Tobacco (*Nicotiana tabacum* cv. K326) obtained from our laboratory, was transformed via an *Agrobacterium*-mediated method using a leaf disc [44]. The shoots were formed in differentiation Murashige and Skoog (MS) medium with 0.5 mg/L indole-3-acetic acid, 2.0 mg/L 6-benzyladenine, 100 mg/L cefotaxim, 200 mg/L timentin and 25 mg/L hygromycin. Then, the shoots were excised and transplanted to root induction medium containing 100 mg/L cefotaxim, 200 mg/L timentin, and 25 mg/L hygromycin. Explants with well-developed roots were transferred into pots containing sterilised vermiculite and grown in a growth chamber at a temperature of 25 ± 2 °C under a 16-h light period (Red and blue LED, 200 μmol m^−2^ s^−1^). The plants were watered with half-strength Hoagland nutrient solution every two weeks. The transformation of these plants was verified by PCR analysis. 

The primary transformants (T_0_) were allowed to self-fertilise, and seeds from the T_1_ generation were used. Two *TaGS1* transformant lines and two *TaGS2* transformant lines were chosen for further analysis. The T_1_ seeds were soaked in 75% ethanol for 4 min, followed by 6.25% sodium hypochlorite for 10 min and then washed five times in sterile water. The treated seeds were sowed in MS medium with 20 mg/L hygromycin and then grown in a growth chamber at a temperature of 25 ± 2 °C under a 16-h light period (Incandescent lamp, 50 µmol m^−2^ s^−1^). After 10 days, some hygromycin-resistant seedlings were transferred to MS medium and maintained in a vertical position for 10 days. Other hygromycin-resistant seedlings were transferred to sterilised vermiculite. The plants were further grown and watered with modified Hoagland nutrient solution (containing 1 mM NH_4_NO_3_, 5 mM KNO_3_, 4 mM Ca(NO_3_)_2_, 1 mM KH_2_PO_4_, 2 mM MgSO_4_, 20 µM Fe-EDTA, 6.7 µM MnSO_4_, 0.32 µM CuSO_4_, 0.77 µM ZnSO_4_, 46µ M H_3_BO_3_, 0.5 µM H_2_MoO_4_, 0.2 µM CoCl_2_, 5 µM KI) in a growth chamber at a temperature of 25 ± 2 °C under a 16-h light period (Red and blue LED, 200 μmol m^−2^ s^−1^) for 9 weeks. After the first 9 weeks of growing with optimal nitrogen nutrition, plants were divided into two sets: those sub-irrigated with the same modified Hoagland nutrient solution containing sufficient nitrogen (N+), or those with modified Hoagland nutrient solution without a nitrogen source (N−) (containing 5 mM KCl, 4 mM CaCl_2_, 1 mM KH_2_PO_4_, 2 mM MgSO_4_, 20 µM Fe-EDTA, 6.7 µM MnSO_4_, 0.32 µM CuSO_4_, 0.77 µM ZnSO_4_, 46 µM H_3_BO_3_, 0.5 µM H_2_MoO_4_, 0.2 µM CoCl_2_, 5 µM KI) and grew for an additional 7 weeks. 

After 9 + 7 weeks, upper, middle and lower leaves were harvested individually. The top fully expanded leaf is considered the threshold. The second and third leaves above this threshold are the upper (new) leaves; the second leaf beneath this threshold is the middle (functional) leaf; leaves near the bottom of the plant are the lower (senescing) leaves (Figure S2). The harvested leaves and roots were immediately frozen and ground in liquid nitrogen, and then the fine homogeneous powder was placed in an Eppendorf tube and stored at −80 °C for further experiments. After harvesting, the leaf area and fresh and dry weights were measured.

### 2.3. RNA Isolation and RT-qPCR Analysis

Total RNA was extracted from the leaves using TRIzol Reagent (Ambion; Thermo Scientific, Waltham, MA, USA), in accordance with the manufacturer’s instructions. The cDNA was synthesised using the First Strand cDNA Synthesis Kit (Thermo Scientific). Quantitative PCR (qPCR) was performed on an iQ5 Multicolor Real-Time PCR (RT-PCR) Detection system (Bio-Rad, Hercules, CA, USA). The qPCR mix was composed of 10 µL AceQ qPCR SYBR Green Master Mix (Vazyme), 9 µL diluted cDNA 1:10 (*v*/*v*), and 1 µL 10 µM forward and reverse primers. All the primers (Sangon) used are shown in Table S2. Reactions proceeded according to the following program: 95 °C for 5 min, followed by 40 cycles of 95 °C for 10 s, 60 °C for 20 s, and 72 °C for 20 s. Fluorescence readings were taken during the elongation step (72 °C). Melting curves were obtained from 60 to 95 °C with a 1 °C increase every 10 s. The relative expression levels of the genes were calculated using the Actin gene as an internal control.

### 2.4. GS Activity Assay and Western Blotting

The fine homogeneous powder obtained from the harvested leaves (0.5 g) was used and mixed with 0.8 mL extraction buffer (100 mM Tris, 1 mM EDTA, 1 mM MgCl_2_, 1 mM Phenylmethanesulfonyl fluoride (PMFS), and 10 mM β-mercaptoethanol, pH 7.6). The extract was centrifuged at 12,000× *g* and 4 °C for 30 min. The obtained supernatant was then prepared for further experiments. 

The total GS activity was measured in accordance with a method described by Ma, et al. [45]. Protein content was determined using the Nanodrop 2000 spectrophotometer (Thermo Scientific). The component proteins were separated using a discontinuous sodium dodecyl sulphate (SDS)-PAGE system, with a 15% analysing gel and 5% stacking gel, and electrophoresis was performed at room temperature. Proteins were transferred to polyvinylidene difluoride membranes for Western blotting. GS polypeptides were detected using polyclonal antibodies raised against GS1 and GS2 of wheat.

### 2.5. Determination of Free NH_4_^+^, NO_3_^−^, Total Amino Acid, and Total Chlorophyll Levels

The free NH_4_^+^ in leaves was determined using the Berthelot colour reaction method [46] with some modifications. The fine homogeneous powder (~0.2 g) was extracted with 1 mL 3% tricarboxylic acid (TCA) at 25 °C for 5 min with shaking. The extracts were then centrifuged at 20,627× *g* and 25 °C for 15 min. Then, 0.5 mL of the supernatant was mixed with 1.5 mL Solution A (5% NaOH, 0.215% NaClO) and 1.5 mL Solution B (6.2% phenol, 0.25‰ sodium nitroprusside) and incubated at 37 °C for 20 min. Finally, the absorbance at 625 nm was determined by UV1000 (AOE Instruments Co., Ltd., Shanghai, China), and NH_4_^+^ contents were calculated from the standard curve of (NH_4_)_2_SO_4_. 

The nitrate in leaves was determined by salicylic acid colorimetry [47] with some modifications. The fine homogeneous powder (~0.2 g) was extracted with 1 mL 3% TCA at 25 °C for 30 min with shaking. The extracts were then centrifuged at 20,627× *g* and 25 °C for 10 min. Next, 50 µL supernatant were mixed with 50 µL 3% TCA and 150 µL salicylic acid solution (5 g salicylic acid, 100 mL sulfuric acid) and incubated at 25 °C for 20 min. Finally, 3.5 mL 8% NaOH were added. After cooling, the absorbance at 410 nm was determined by UV1000 (AOE), and nitrate levels were calculated from the standard curve of KNO_3_.

Total amino acids were measured by ninhydrin colorimetry with some modifications. The fine homogeneous powder (about 0.2 g) was extracted with 1 mL 10% acetic acid at 25 °C for 10 min with shaking. The extracts were centrifuged at 20,627× *g* and 25 °C for 10 min. Then, 150 µL of the supernatant were mixed with 2 mL acetate buffer (pH 5.4), 3 mL ninhydrin solution, and 0.1 mL 0.1% ascorbic acid and heated at 100 °C for 15 min. After cooling, the absorbance at 580 nm was determined by UV1000 (AOE), and the amino acid content was calculated from the standard curve of leucine.

Total chlorophyll was extracted from the fine homogeneous leaf powder (~0.2 g) using 1.5 mL 80% acetone at 25 °C for 10 min with shaking. The extracts were centrifuged at 13,201× *g* and 25 °C for 15 min. Then, 1 mL supernatant was mixed with 3 mL 80% acetone. The absorbance at 664 and 647 nm was determined by UV1000 (AOE), and the total chlorophyll content was calculated as described previously [48]. 

### 2.6. Statistics

All data represent mean ± standard deviation (SD) of three biological replicates. The data sets were analysed using Microsoft Excel (2016, Microsoft, Redmond, WA, USA), the data were statistically analysed using SPSS version 13.0 (IBM, Chicago, IL, USA). One-way analysis of variance with a Duncan post hoc test was used for testing differences.

## 3. Results

### 3.1. Identification of Transgenic Tobacco

Transformation vectors containing *TaGS1* or *TaGS2* were constructed as shown in Figure S1. The expression of *TaGS1* and *TaGS2* was under the control of a super-promoter [38]. The ligated gene fragments were transformed into tobacco by an *Agrobacterium*-mediated method using a leaf disc. The transgenic lines containing *TaGS1* were designated GS1-TR, while those containing *TaGS2* were designated GS2-TR. Six GS1-TR lines and six GS2-TR lines were confirmed by PCR. Four independent transformed lines, GS1-TR1, GS1-TR2, GS2-TR1 and GS2-TR2, were used for the nitrogen starvation experiment.

### 3.2. Characteristics of GS Expression in GS1-TR and GS2-TR

**Figure 1:**
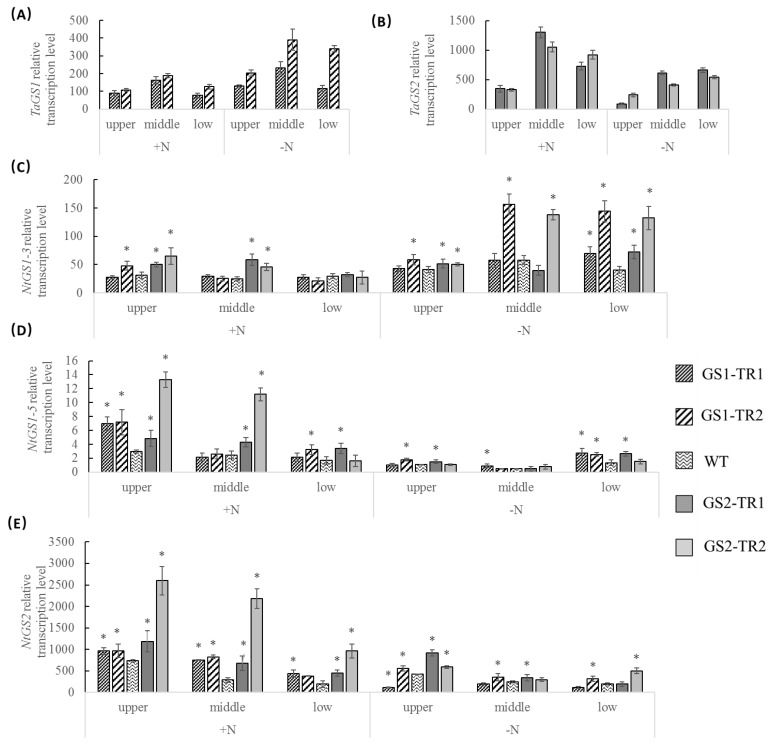
Relative transcription levels of *Triticum aestivum* glutamine synthetase (GS) *TaGS1* (**A**), *TaGS2* (**B**), *Nicotiana tabacum* glutamine synthetase (GS) *NtGS1-3* (**C**), *NtGS1-5* (**D**) and *NtGS2* (**E**) in the upper, middle, and lower leaves of transgenic plants grown under nitrogen-sufficient (N+) and nitrogen-starvation (N−) conditions. Data are means of three independent biological replicates ± standard deviation (SD). Asterisks indicate that the data is significantly different (*p* < 0.05) from the data of wild type (WT) plants.

The mRNA levels of *TaGS1, TaGS2*, *NtGS1-3*, *NtGS1-5* and *NtGS2* were monitored by reverse transcription quantitative PCR (RT-qPCR). The transcription of *TaGS1* were about two times of *NtGS1-3* and about 50 times of *NtGS1-5* in the leaf of *TaGS1* transformant lines, while the transcription of *TaGS2* were less than that of *NtGS2* in the leaf of *TaGS2* transformant lines. Under nitrogen-sufficient conditions, the transcription of *TaGS1*, *NtGS1-3* and *NtGS1-5* was much lower than that of *TaGS2* and *NtGS2* in leaves of different rank, and the transcription of *TaGS2* was the highest in the middle leaves, higher in the lower leaves and the lowest in the upper leaves, while the transcription of *NtGS2* was highest in the upper leaves, higher in the middle leaves and lowest in the lower leaves, and the transcription of *NtGS2* in the *TaGS* transformants was higher than that in the WT, especially in the GS2-TR2 (Figure 1). Under nitrogen starvation, the transcription of *TaGS1* and *NtGS1-3* was increased significantly in the leaves of different rank, especially in the middle and lower leaves of GS1-TR2, while the transcript of *TaGS2*, *NtGS2* and *NtGS1-5* were decreased significantly in the different rank leaves (Figure 1). In a word, the leaf age and nitrogen condition affected *TaGS1* and *TaGS2* expression in transgenic tobacco, and *TaGS1* expressed more while *TaGS2* expressed less compared with the background *NtGS1* (*NtGS1-3*, *NtGS1-5)* and *NtGS2* respectively.

**Figure 2:**
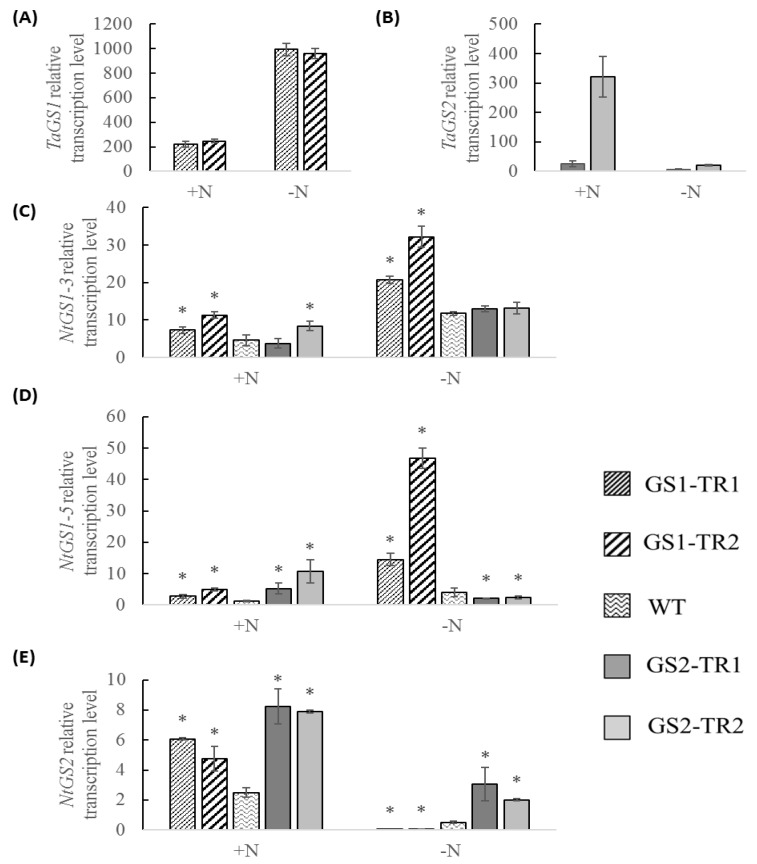
Relative transcription levels of *TaGS1* (**A**), *TaGS2* (**B**), *NtGS1-3* (**C**), *NtGS1-5* (**D**) and *NtGS2* (**E**) in the root of transgenic plants grown under nitrogen-sufficient (N+) and nitrogen-starvation (N−) conditions. Data are means of three independent biological replicates ± SD. Asterisks indicate that the data is significantly different (*p* < 0.05) from the data of WT plants.

Since GS plays important functions in plant roots of plants, the expression of GS in roots was also monitored. In *TaGS1* transformant lines, *TaGS1* overexpressed observably, about 20 times that of *NtGS1-3* under N+ treatment, and about 30 times that of *NtGS1-3* under N− treatment; the transcription of *TaGS1* under N− treatment was about five times that under N+ treatment (Figure 2A,C). In *TaGS2* transformant lines, *TaGS2* was also observably overexpressed, about 4–50 times that of *NtGS2* under N+ treatment, and about 3–7 times that of *NtGS2* under N− treatment; the transcription of *TaGS2* under N+ treatment was about 4–15 times that under N− treatment (Figure 2B,E). Compared with the WT, the transcription of *NtGS1-3*, *NtGS1-5* and *NtGS2* in *TaGS* transformants were up-regulated under nitrogen-sufficient conditions; under nitrogen-starvation conditions, the transcription of *NtGS1-3* and *NtGS1-5* were up-regulated but the transcription of *NtGS2* were down-regulated in *TaGS1* transformants. Furthermore, the transcription of *NtGS1-5* was down-regulated but the transcription of *NtGS2* were up-regulated in *TaGS2* transformants (Figure 2C–E).

### 3.3. Characteristics of GS Subunit Expression in GS1-TR and GS2-TR

**Figure 3:**
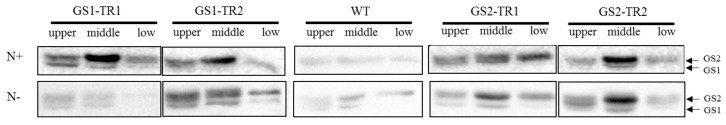
GS expression in the upper, middle and lower leaves of GS1-TR, GS2-TR, and the WT grown under a N+ or N− regime. Western blot analysis using various antibodies against wheat cytosolic GS1 and chloroplastic GS2.

The molecular masses of the NtGS1and NtGS2 subunits are approximately 39 and 44 kDa, respectively [49], which are similar to those of the TaGS1 (39 kDa) and TaGS2 (42 kDa) subunits. Therefore, NtGS1 and TaGS1, NtGS2 and TaGS2 showed the same migration pattern on SDS-PAGE, respectively. Because the GS of wheat and tobacco had high amino acid sequence similarity, GS antibodies could not distinguish GS isozymes. Under nitrogen-sufficient conditions, four transformants showed significantly higher levels of GS1 and GS2 subunits compared to WT. The contents of GS2 subunit were highest in middle leaves, higher in upper leaves and lowest in the lower leaves, and the contents of GS1 subunit were very low in GS1-TR2 and GS2-TR, but no GS1 subunit content was found in the leaves of WT (Figure 3). It was obvious that the expression of GS1 in the leaf was inhibited under nitrogen-sufficient conditions (Figure 3). Under nitrogen starvation, compared with the WT, GS2-TR had much higher levels of GS2 subunits in the leaves of different rank, especially in the middle leaves, and also had higher levels of GS1 subunits in the middle leaves (Figure 3). Compared with WT, the GS1-TR2 showed significantly higher levels of GS1 and GS2 subunits in the leaves of different rank, but the GS1-TR1 showed lower levels of GS1 and GS2 subunits (Figure 3). 

**Figure 4:**
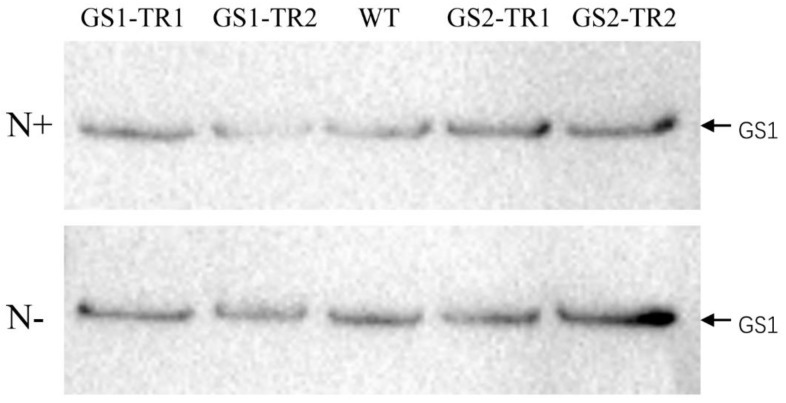
GS expression in the root of GS1-TR, GS2-TR, and the WT grown under a N+ or N− regime. Western blot analysis using various antibodies against wheat cytosolic GS1 and chloroplastic GS2.

In the roots, GS1 was the main subunit and no GS2 polypeptide was detected in WT and *TaGS* transformants. Compared with the WT, *TaGS* transformants had higher levels of GS1 subunits (Figure 4). Under nitrogen starvation, the contents of GS1 subunit were higher than those under nitrogen-sufficient conditions (Figure 4).

### 3.4. Characteristics of GS Activity in GS1-TR and GS2-TR

**Figure 5:**
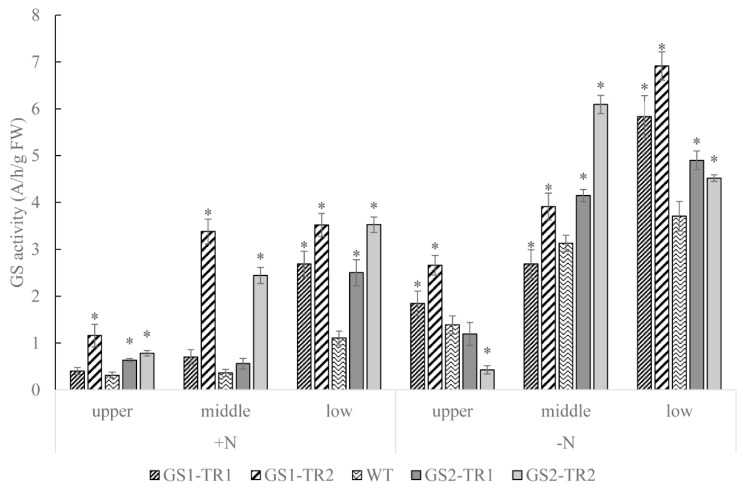
The GS activity in the upper, middle and lower leaves of GS1-TR and GS2-TR compared with that in the WT grown under a N+ or N− regime. Each value is the mean ± standard error of three replicates. Asterisk indicate that the date is significantly different (*p* < 0.05) from the data of WT plants.

Under nitrogen-sufficient conditions, compared with the WT, GS1-TR and GS2-TR had higher total GS activity than those in the leaves of different rank, except for the upper leaves of GS1-TR1. Under nitrogen-starvation conditions, the total GS activity of WT and *TaGS* transformants was higher than those under nitrogen-sufficient conditions, except in the upper leaves of GS2-TR2. Compared with the WT, *TaGS* transformants showed significantly higher total GS activity in the leaves of different rank, except for the middle leaves of GS1-TR1 and the upper leaves of GS2-TR1 and GS2-TR2 (Figure 5).

### 3.5. Characteristics of Nitrogen Metabolic Status in GS1-TR and GS2-TR 

In plants, NO_3_^−^ is the major storage form of nitrogen [50]. Under nitrogen starvation, the NO_3_^−^ content in leaves was only about 20% of that under nitrogen sufficiency (Figure S3), showing that the nitrogen storage in leaves was dramatically reduced under nitrogen starvation. 

**Figure 6:**
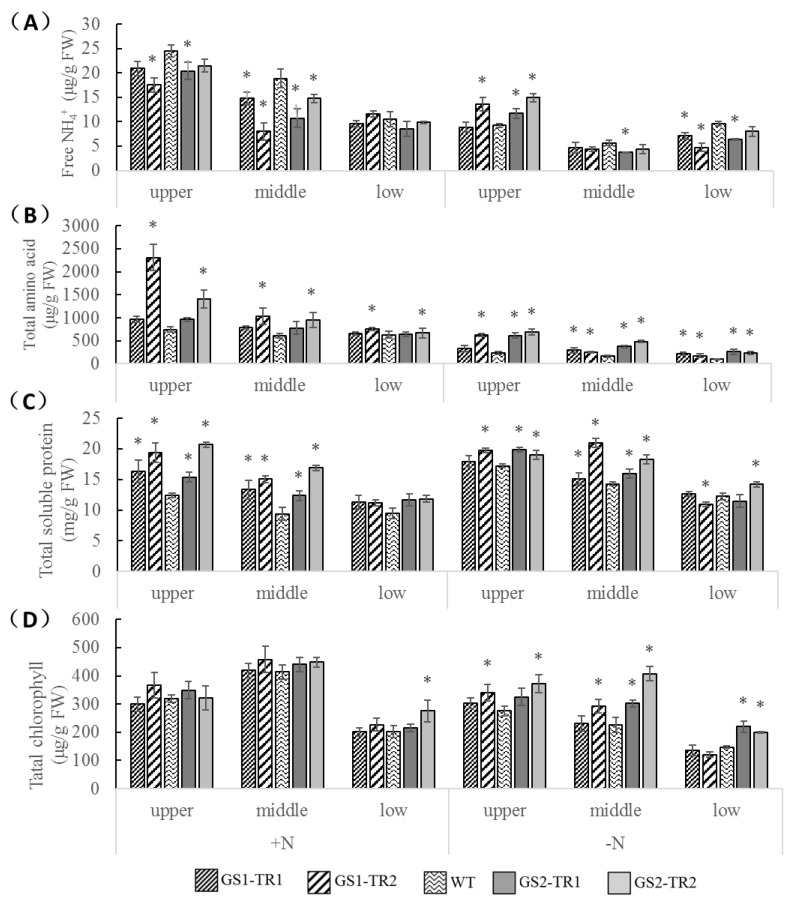
Nitrogen metabolite levels in the upper, middle, and lower leaves of GS1-TR, GS2-TR, and the WT plants grown under nitrogen-sufficient and nitrogen-starvation conditions. The free NH_4_^+^ (**A**), total free amino acid (**B**), total soluble protein (**C**) and total chlorophyll (**D**) content were determined. Each value is shown as the mean ± standard error of three replicates. Asterisks indicate that the date is significantly different (*p* < 0.05) from the data of WT plants.

Under nitrogen-sufficient conditions, the NH_4_^+^ levels of the two transformants were significantly decreased in the upper and middle leaves compared with those in the WT (Figure 6A), while the amino acid and total soluble protein levels were significantly increased (Figure 6B,C). These results indicate that *TaGS* overexpression may improve the efficiency of NH_4_^+^ assimilation into amino acids and protein. However, the chlorophyll levels (Figure 6D) of the four transformants showed non-significant differences compared with those in the WT, except for low leaves of GS2-TR2. 

Under nitrogen starvation, NH_4_^+^ is generated mainly from the self-metabolism process of the plants and degradation of nitrogenous substances such as proteins and chlorophyll. The NH_4_^+^ contents in the lower leaves of GS1-TR1, GS1-TR2, GS2-TR1 and the middle leaves of GS2-TR1 were significantly lower than that in the WT, and the NH_4_^+^ content was lower than that in the respective leaves under nitrogen-sufficient conditions (Figure 6A), indicating that overexpression of *TaGS1* or *TaGS2* can improve NH_4_^+^ assimilation. Interestingly, higher NH_4_^+^ content was observed in the upper leaves of GS1-TR2, GS2-TR1 and GS2-TR2, compared with WT; these results may be related to the strong self-metabolism process and lower GS activity of upper leaves. Amino acid contents differed significantly in the leaves of different rank among GS1-TR, GS2-TR and WT plants with the following order: GS2-TR > GS1-TR > WT (Figure 6B). For GS1-TR2, the contents of amino acid, chlorophyll and soluble protein in upper and middle leaves were significantly higher than those in WT, but the amino acid content was significantly higher and the soluble protein content was significantly lower in the lower leaves. The chlorophyll and soluble protein contents of the GS2-TR leaves were higher than that of WT leaves (Figure 6C,D). These results suggest that overexpression of *TaGS1* or *TaGS2* can improve nitrogen reassimilation in different ways, but GS2-TR is more efficient at reassimilating nitrogen than is GS1-TR.

### 3.6. Phenotypes of GS1-TR and GS2-TR 

**Figure 7:**
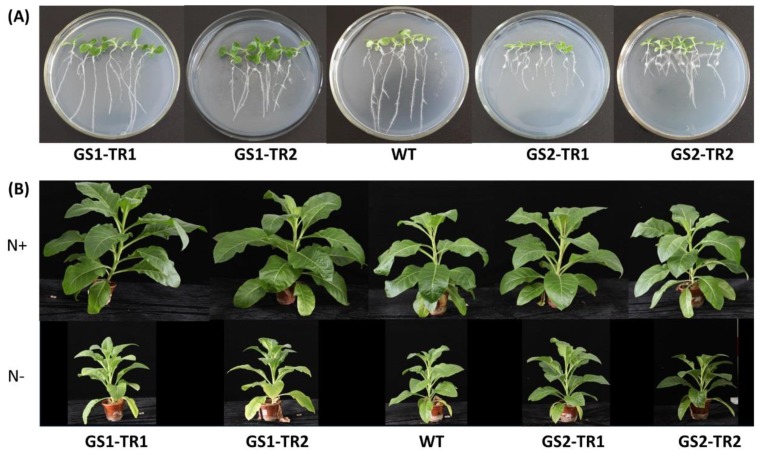
Qualitative growth phenotypes of transgenic and WT plants. (**A**) Comparison of seedlings from the WT and transgenic tobacco GS1-TR and GS2-TR. The plants were germinated and grown for 20 days in Murashige and Skoog (MS) medium as described in Materials and Methods section. (**B**) The phenotype of transgenic tobacco GS1-TR and GS2-TR, compared with that of the WT, after 7 weeks of growth under nitrogen-sufficient (N+) and nitrogen-starvation (N−) conditions.

Compared with the WT, GS2-TR seedlings had more but shorter lateral roots, and GS1-TR seedlings had longer lateral roots (Figure 7A and Table S3), suggesting that overexpression of *TaGS1* and *TaGS2* can stimulate the lateral root formation of transgenic tobacco. With regard to the shoots, GS1-TR exhibited a larger leaf area than WT during the seedling stage (Figure 7A, which is consistent with the findings of Oliveira, Brears, Knight, Clark and Coruzzi [27].

Under nitrogen-sufficient conditions, GS1-TR showed significantly higher leaf area and plant height than those in WT, but GS2-TR showed a similar phenotype to the WT, with no difference in plant height, leaf area and plant dry weight (Figure 7B, Table 1). Under nitrogen starvation, GS1-TR showed an improved phenotype compared to WT, i.e., a higher plant height and greener and larger upper leaves, and GS2-TR exhibited greener and larger leaves (Figure 7B). The plant height, dry weights and the leaf area of GS1-TR increase significantly compared with the WT, except for the plant height of the GS1-TR1. For GS2-TR, the plants were smaller than WT, especially for GS2-TR2. However, the plant dry weights and the leaf area of GS2-TR did not significantly reduce, and the leaf area of GS2-TR1 increased significantly (Figure 7B, Table 1). These results suggest that overexpression of *TaGS1* or *TaGS2* can improve tobacco growth, and when nitrogen is a limiting factor of tobacco growth, overexpression of *TaGS1* or *TaGS2* improves the tolerance to nitrogen stress in different ways.

**Figure 8:**
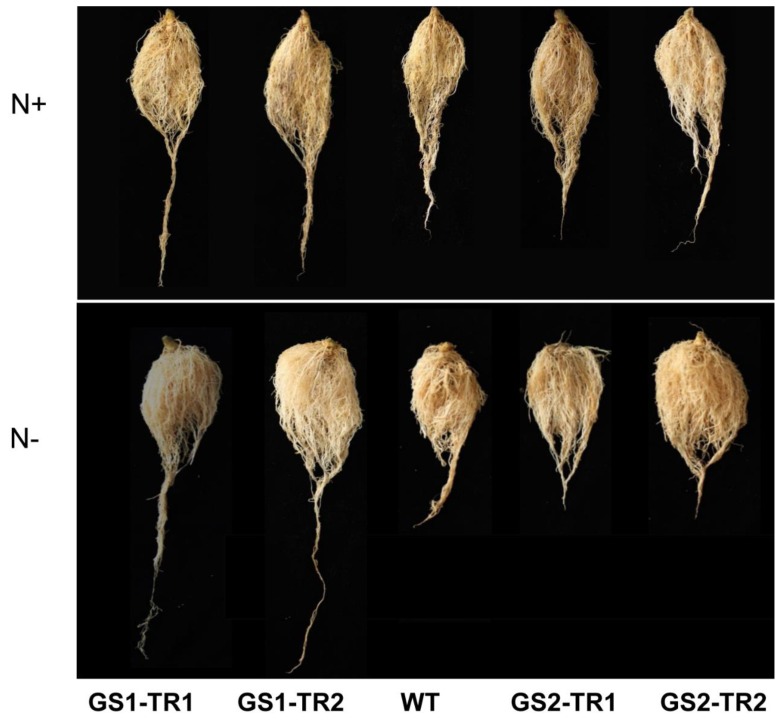
The root phenotype of transgenic tobacco GS1-TR and GS2-TR, compared with that of the WT, after 7 weeks of growth under nitrogen-sufficient (N+) and nitrogen-starvation (N−) conditions.

The roots are central to the acquisition of nitrogen and play a central role for NUE. Compared with the WT, GS1-TR had longer root length and significantly higher root dry weight, but GS2-TR showed a similar root length to the WT, and only GS2-TR2 had significantly higher root weight (Figure 8 and Table 1). Under nitrogen starvation, the dry weight of GS1-TR was higher than that under nitrogen-sufficient conditions; in addition, the root length of GS1-TR increased under nitrogen starvation (Figure 8 and Table 1). 

## 4. Discussion

GS has an important role in the assimilation of inorganic nitrogen. For decades, in order to improve nitrogen use efficiency, overexpression of GS has been investigated in numerous cases. However, the outcome has generally been inconsistent [5,23]. Therefore, it is necessary to parse the reasons if we want to improve NUE by overexpressing GS. The way to overexpress GS has mainly been based on the use of constitutive promoters [21,25,27,28,29,51]. The expression of endogenous GS is regulated by complex mechanisms [5] at pre-transcriptional, transcriptional and post-transcriptional levels to adapt to plant growth and environmental conditions. This multilevel regulation of GS can potentially interfere with the expression of GS driven by a constitutive promoter. 

The super-promoter is a constitutive promoter; it can efficiently drive transgene expression in leaf and root of tobacco [38]. In this study, under nitrogen-sufficient conditions, the leaves of GS2-TR showed high accumulation of the *TaGS2* transcript, while those of GS1-TR showed a low *TaGS1* transcript levels (Figure 1A,B). However, under nitrogen starvation, the *TaGS1* transcript level increased in the leaves of different rank, but the *TaGS2* transcript level decreased in leaves of different rank (Figure 1A,B). Furthermore, the transcription of *TaGS1* in the roots was much higher than that of *TaGS2* in N− and N+ treatment (Figure 2). These results would suggest that although *TaGS1* and *TaGS2* were driven by a constitutive super-promoter, their expression was regulated by nitrogen condition and organs. 

Under nitrogen-sufficient conditions, the transcript level of *TaGS2* in the middle leaves was about 2.5-fold that in the upper leaves (Figure 1B). Furthermore, increases in GS polypeptide content and activity were detected in the leaves of the four transformants, especially in the middle and lower leaves (Figure 3 and Figure 4). Under nitrogen starvation, the *TaGS1* and *TaGS2* transcript levels were highest in the middle leaves (Figure 1A,B). Moreover, GS activity of the four transformants increased significantly in the middle and lower leaves compared with the activity of the WT (Figure 4). These results suggest that although *TaGS1* and *TaGS2* were driven by a constitutive super-promoter, the expression of *TaGS1* and *TaGS2* was regulated by leaf age, and overexpression of *TaGS1* or *TaGS2* can improve the GS activity and polypeptide content. 

*GS1* and *GS2* have different functions in plant growth; accordingly, the genes expression must be regulated in different manners to match genes different roles. Which is even more intriguing, given that nitrogen supply and leaf age had similar effect on the expression of endogenous genes of *NtGS1-3* and *NtGS2* and exogenous genes of *TaGS1* and *TaGS2* in the transcript levels (Figure 1). Under nitrogen-starvation conditions, the nitrogen in senescing (lower) leaves becomes very precious for transferral to the developing leaves. Improving the transcript level of *TaGS1* and *NtGS1-3* can facilitate nitrogen transport from senescing leaves to newly budded leaves (upper) to enable plant survival under nitrogen stress. When inorganic nitrogen is not available, the content of NH_4_^+^ derived from nitrate reduction decreases, and this part of the ammonia assimilation process would decrease correspondingly, leading to decreased transcript levels of *TaGS2* and *NtGS2* in the middle and lower leaves (functional leaves) (Figure 1B,E). In this regard, exogenous gene expression is dependent not only on the constitutive promoter, but also on the gene itself, the plant developmental stage and the environment. 

Due to the CDS of *TaGS1* or *TaGS2* being driven by a constitutive super-promoter, there is no regulation of transcriptional level. Our data suggests that there may be a post-transcriptional regulation that plays a major role in controlling the accumulation of the *TaGS1* and *TaGS2* in tobacco. Numerous studies have implicated microRNAs (miRNAs) as key regulators of post-transcriptional gene expression [52,53]. miRNAs are small ~21–22 nt molecules that play critical roles in various developmental, stress and signalling responses [54]. Recent studies have examined the changes in expression levels of miRNAs in response to nitrogen starvation in maize [55,56], rice [57] and *Arabidopsis* [58,59]. GS is the key enzyme in nitrogen metabolism, and the expression of GS is regulated by nitrogen supply and plant developmental stage [33,34]. Therefore, we presumed that the transcription level of *TaGS1* or *TaGS2* might be negatively regulated by miRNAs in the post-transcriptional level.

In addition to nitrogen supply and leaf age, other factors can also affect the expression of *GS*. In sorghum, two different GS1 isoforms were induced by NH_4_^+^ but not by nitrate [60]. In *Arabidopsis*, red light increased the expression of *GS2*, but far-red light decreased *GS2* expression [61]. In peas, high CO_2_ content inhibited the expression of *GS2* [62]. We presumed that these factors may also affect the expression of *GS*, even when driven by a constitutive promoter.

In roots, the transcription of *TaGS1* was much higher than that of *TaGS2*, accordingly more GS1 subunit (no GS2) was detected in *TaGS* transformants than in WT (Figure 2 and Figure 4). These results suggest that the translation of *TaGS2* was inhibited in the root. Therefore, the change of root phenotype in *TaGS2* transformants may be mainly caused by the change of *NtGS1-3* and *NtGS1-5*. In *TaGS1* transformants, the root length and root dry weight and the expression of endogenous gens of *NtGS1-3* and *NtGS1-5* and exogenous gens of *TaGS1* increased compared to WT (Figure 2 and Figure 8, Table 1 and Table S3). These results show that *TaGS1*, *NtGS1-3* and *NtGS1-5* can promote root growth.

The assimilation of inorganic nitrogen that takes place through the GS/GOGAT pathway requires carbon skeletons, reducing power and ATP, which are provided directly or indirectly by photosynthesis [63,64]. Meanwhile, a high photosynthetic rate also requires a sufficient nitrogen supply. In C3 plants, GS2 plays an important role in the reassimilation of NH_4_^+^ derived from photorespiration [11]. Compared with the WT, the leaves of GS2-TR had lower free NH_4_^+^ (Figure 6A) and higher amino acid (Figure 6B) and soluble protein (Figure 6C) levels under nitrogen-starvation conditions. These results suggest that *TaGS2* overexpression can improve nitrogen assimilation. Therefore, the chlorophyll level remained higher in GS2-TR than that in WT plants (Figure 6D), leading to a higher photosynthetic rate (Figure S4). 

During leaf senescence, nitrogenous compounds are used as nutrient sources to build new organs [20,65]. GS1 plays a key role in nitrogen remobilisation in this process [5,10]. However, the ability of GS1 to transport nitrogen seems to be insufficient [66]. In our study, when the middle and lower leaves of GS1-TR began to senesce under nitrogen starvation (Figure 7B), the levels of *TaGS1* and *NtGS1-3* expression and GS activity were increased in GS1-TR (Figure 1A,C and Figure 4), resulting in a higher content of free amino acids (Figure 6B), the main form of nitrogen translocation in the senescent leaves [67]. Additionally, the upper leaves had higher levels of chlorophyll and soluble protein than those of the WT (Figure 6C,D). These results indicate that *TaGS1* overexpression may improve the efficiency of nitrogen remobilisation from senescent leaves to developing ones. 

Under nitrogen starvation, *TaGS1* and *TaGS2* transgenic plants had higher GS activity and a better nitrogen metabolism status than those of WT, suggesting that applying less nitrogen to GS transgenic plants may be an effective way to increase nitrogen use by improving the ability to assimilate nitrogen or the efficiency of nitrogen remobilisation. Sun, Huang and Su [30] found that *GS1* and *GS2* overexpression simultaneously induced tolerance to nitrogen starvation in transgenic plants. Therefore, based on these complementary functions of *TaGS1* and *TaGS2*, concurrent overexpression of *GS1* and *GS2* may be an effective method to improve the efficiency of nitrogen use.

## Figures and Tables

**Table 1:** Plant height, leaf area, dry weight (DW) and root dry weight of GS1-TR, GS2-TR and WT plants grown under nitrogen-sufficient (N+) and nitrogen-starvation (N−) conditions.

	N+				N−			
	Plant Height (cm)	Leaf Area (cm^2^)	Plant DW (g)	Root DW (g)	Plant Height (cm)	Leaf Area (cm^2^)	Plant DW (g)	Root DW (g)
GS1-TR1	35.5 ± 1.1 *	3732 ± 449 *	5.2 ± 0.8	0.64 ± 0.09 *	21.2 ± 2	1392 ± 66 *	3.9 ± 0.4 *	0.72 ± 0.03 *
GS1-TR2	33 ± 1.5 *	3340 ± 323 *	6 ± 1.2	0.65 ± 0.07 *	25.5 ± 2.3 *	1552 ± 75 *	5.2 ± 0.5 *	0.89 ± 0.04 *
WT	26 ± 2.3	2331 ± 317	4.2 ± 0.2	0.51 ± 0.02	20.3 ± 1	1190 ± 53	3.1 ± 0.2	0.55 ± 0.04
GS2-TR1	27.8 ± 1.9	2885 ± 241	5.3 ± 1.2	0.55 ± 0.07	19 ± 1.1	1442 ± 126 *	3.5 ± 0.1	0.59 ± 0.02
GS2-TR2	27.2 ± 2.5	3093 ± 299	5.1 ± 0.6	0.65 ± 0.04 *	14.8 ± 0.2 *	1338 ± 42	3.6 ± 0.2	0.67 ± 0.01 *

Note: Each value is shown as the mean ± standard error of three replicates. Asterisk indicate that the date is significantly different (*p* < 0.05) from the data of WT plants.

## Supplementary Materials

The following are available online at http://www.mdpi.com/2073-4425/9/8/406/s1, **Figure S1**: Recombinant vector containing a derivative of the super-promoter, GS1 or GS2, and the NOS terminator between the right (RB) and left borders (LB) of the T -DNA. The hygromycin resistance gene (hyg) was located between the 35S promoter and poly-A tail. **Figure S2**: Leaf strata designation of tobacco plants used in this study. **Figure S3**: The NO_3_^−^ content in the upper, middle, and lower leaves of GS1-TR, GS2-TR, and the WT plants grown under nitrogen sufficient and nitrogen-starvation conditions. Each value is shown as the mean ± standard error of three replicates. Asterisk indicate that the date is significantly different (*p* < 0.05) from the data of WT plants. **Figure S4**: CO_2_ response curve (A) and light response curve (B) of photosynthesis in the middle leaves from TR-GS1, TR-GS2, and WT plants grown under optimum nitrogen and nitrogen-starvation conditions. **Table S1**: The primers used to amplify the full cDNA of TaGS1 and TaGS2 from wheat. **Table S2**: The primers used in quantitative RT-PCR analysis of transgenic lines and Wild Type. **Table S3**: Total root length and lateral root number of seedlings from GS1-TR, GS2-TR, and WT plants.
