# Supplementary material for: Nitrogen Supply and Leaf Age Affect the Expression of TaGS1 or TaGS2 Driven by a Constitutive Promoter in Transgenic Tobacco
Source: Genes (Basel). 2018 Aug 10;9(8):406. doi: 10.3390/genes9080406 (PMC6115907; doi:10.3390/genes9080406)
Supplement: Supplementary file 1 [file genes-09-00406-s001.zip › Supplementary/Figure S1.pdf]

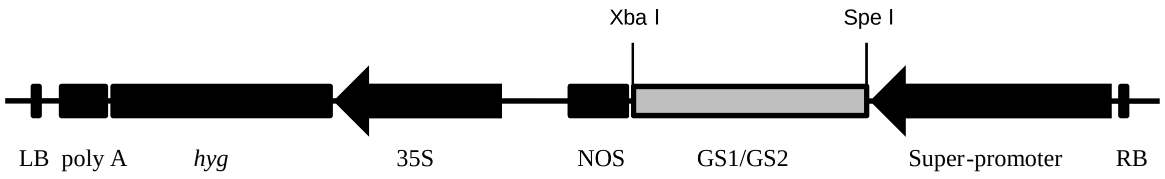

Figure S1 Recombinant vector containing a derivative of the super-promoter, GS1 or GS2, and the NOS terminator between the right (RB) and left borders (LB) of the T-DNA. The hygromycin resistance gene (hyg) was located between the 35S promoter and poly-A tail.
