# Supplementary material for: Nitrogen Supply and Leaf Age Affect the Expression of TaGS1 or TaGS2 Driven by a Constitutive Promoter in Transgenic Tobacco
Source: Genes (Basel). 2018 Aug 10;9(8):406. doi: 10.3390/genes9080406 (PMC6115907; doi:10.3390/genes9080406)
Supplement: Supplementary file 1 [file genes-09-00406-s001.zip › Supplementary/Figure S3.pdf]

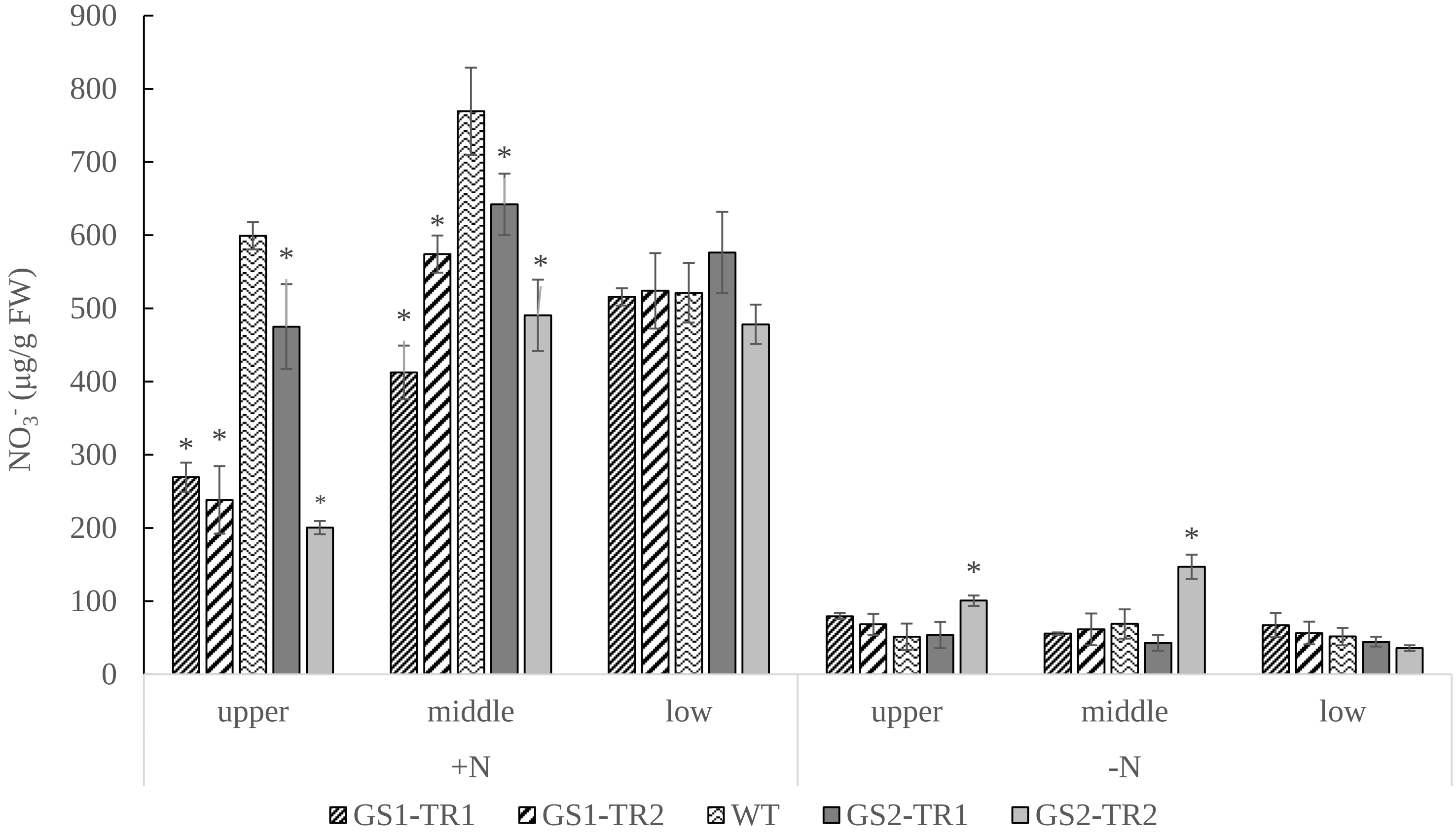

Figure S3 The NO_3^- content in the upper, middle, and lower leaves of GS1-TR, GS2-TR, and the WT plants grown under nitrogen-sufficient and nitrogen-starvation conditions. Data are means of three independent biological replicates ± SD. Asterisk indicate that the data is significantly different ($p<0.05$) from the data of WT plants.
