# Supplementary material for: Nitrogen Supply and Leaf Age Affect the Expression of TaGS1 or TaGS2 Driven by a Constitutive Promoter in Transgenic Tobacco
Source: Genes (Basel). 2018 Aug 10;9(8):406. doi: 10.3390/genes9080406 (PMC6115907; doi:10.3390/genes9080406)
Supplement: Supplementary file 1 [file genes-09-00406-s001.zip › Supplementary/Figure S4.pdf]

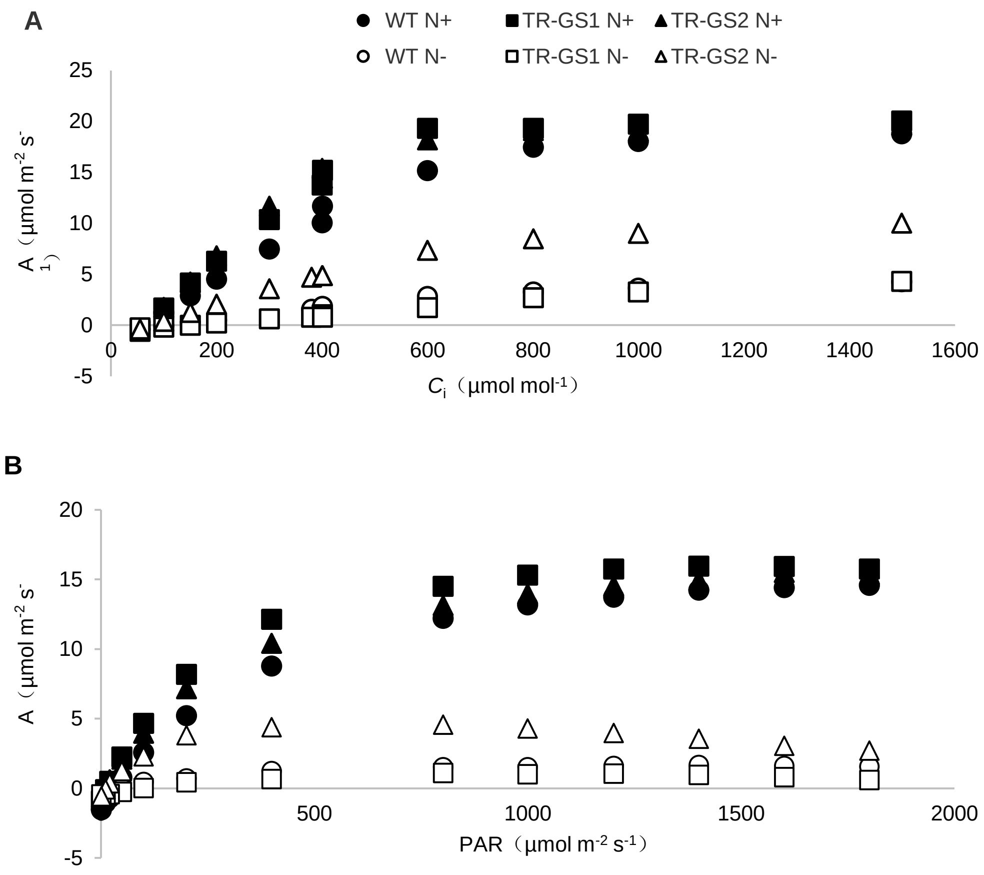

Figure S4 CO_2 response curve（A）and light response curve（B） of photosynthesis in the middle leaves from TR-GS1, TR-GS2, and WT plants grown under optimum nitrogen and nitrogen starvation conditions.
