# Supplementary material for: Nitrogen Supply and Leaf Age Affect the Expression of TaGS1 or TaGS2 Driven by a Constitutive Promoter in Transgenic Tobacco
Source: Genes (Basel). 2018 Aug 10;9(8):406. doi: 10.3390/genes9080406 (PMC6115907; doi:10.3390/genes9080406)
Supplement: Supplementary file 1 [file genes-09-00406-s001.zip › Supplementary/Table S1.docx]

**Table S1** The primers used to amplify the full cDNA of TaGS1 and TaGS2 from wheat

| Gene Name | Primer | Sequence(5'-3') |
| --- | --- | --- |
| *TaGS1* | *TaGS1-F* | CCAAATCGACTCTAGTCTAGAATGGCGCTCCTCACCGATCTCCTC |
|  | *TaGS1-R* | CTCGGTACCGGATCCACTAGTTCAGGGCTTCCACAGGATGGTGGTC |
| *TaGS2* | *TaGS2-F* | CCAAATCGACTCTAGTCTAGAAT GGCGCAGGCGGTGGTG |
|  | *TaGS2-R* | CTCGGTACCGGATCCACTAGTTC ATACCTTCAGCGCCAGCTTCTTG |
