# Supplementary material for: Nitrogen Supply and Leaf Age Affect the Expression of TaGS1 or TaGS2 Driven by a Constitutive Promoter in Transgenic Tobacco
Source: Genes (Basel). 2018 Aug 10;9(8):406. doi: 10.3390/genes9080406 (PMC6115907; doi:10.3390/genes9080406)
Supplement: Supplementary file 1 [file genes-09-00406-s001.zip › Supplementary/Table S2.docx]

**Table S2** The primers used in quantitative RT-PCR analysis of transgenic lines and Wild Type

| Gene Name | Primer | Sequence(5'-3') |
| --- | --- | --- |
| *NtGS1-3* | *NtGS1-3-F* | CGGAGACTGCTAGAGTGGTTGTGT |
|  | *NtGS1-3-R* | GAAGGTGCTGATGTTGGCTGTTT |
| *NtGS1-5* | *NtGS1-5-F* | TCTCACTGGAAGACACGAAACAGC |
|  | *NtGS1-5-R* | CGGATAGGATAGTGGTCTCAGCAA |
| *NtGS2* | *NtGS2-F* | ATGGCAGATGAGAATGACAAAGAGC |
|  | *NtGS2-R* | GTACGGAGTTACATCCAAGTTTAGCAG |
| *TaGS1* | *TaGS1-RT-F* | AACCAATCTCGTTACACCAAATCG |
|  | *TaGS1-RT-R* | GTGCTGGAGCCGTCGAAGTTC |
| *TaGS2* | *TaGS2-RT-F* | AACCAATCTCGTTACACCAAATCG |
|  | *TaGS2-RT-R* | CCAGATCCTCCAACCCAGATGTAC |
| *NtActin* | *NtActin-F* | TACTTACTGAAGCACCCTTGAATCC |
|  | *NtActin-R* | GATCACGACCAGCAAGATCCAAC |
