# Supplementary material for: Nitrogen Supply and Leaf Age Affect the Expression of TaGS1 or TaGS2 Driven by a Constitutive Promoter in Transgenic Tobacco
Source: Genes (Basel). 2018 Aug 10;9(8):406. doi: 10.3390/genes9080406 (PMC6115907; doi:10.3390/genes9080406)
Supplement: Supplementary file 1 [file genes-09-00406-s001.zip › Supplementary/Table S3.docx]

**Table S3** Total root length and lateral root number of seedlings from GS1-TR, GS2-TR, and WT plants.

|  | Total root length (cm) | Lateral root number |
| --- | --- | --- |
| GS1-TR1  GS1-TR2 | 9.6±1.7 *  6.8±1.0 | 3.2±0.4 *  4.2±0.5 * |
| WT | 7.3±0.9 | 2.2±0.4 |
| GS2-TR1  GS2-TR2 | 4.9±0.7 *  5.2±0.3 * | 3.7±0.7 *  3.1±0.6 * |

**Note:** Data are means of three independent biological replicates ± SD. Asterisk indicate that the data is significantly different (p<0.05) from the data of WT plants.
